# Relevance of the KATP channel function for the basal insulin hypersecretion of islets from female NZO mice

**DOI:** 10.1210/jendso/bvag108

**Published:** 2026-05-13

**Authors:** Melissa Asuaje Pfeifer, Joana Brozek, Katharina Grupe, Florian Wanitschke, Stephan Scherneck, Ingo Rustenbeck

**Affiliations:** Institute of Pharmacology, Toxicology and Clinical Pharmacy, Technische Universität Braunschweig, D38106 Braunschweig, Germany; Institute of Pharmacology, Toxicology and Clinical Pharmacy, Technische Universität Braunschweig, D38106 Braunschweig, Germany; Institute of Pharmacology, Toxicology and Clinical Pharmacy, Technische Universität Braunschweig, D38106 Braunschweig, Germany; Institute of Pharmacology, Toxicology and Clinical Pharmacy, Technische Universität Braunschweig, D38106 Braunschweig, Germany; Institute of Pharmacology, Toxicology and Clinical Pharmacy, Technische Universität Braunschweig, D38106 Braunschweig, Germany; PVZ-Center of Pharmaceutical Engineering of the Technische Universität Braunschweig, D38106 Braunschweig, Germany; Institute of Pharmacology, Toxicology and Clinical Pharmacy, Technische Universität Braunschweig, D38106 Braunschweig, Germany; PVZ-Center of Pharmaceutical Engineering of the Technische Universität Braunschweig, D38106 Braunschweig, Germany

**Keywords:** β cells, cytosolic calcium concentration, insulin secretion, KATP channel, membrane potential, type 2 diabetes

## Abstract

**Context:**

The polygenic New Zealand obese (NZO) mouse model displays the characteristics of early type 2 diabetes: fasting hyperglycemia, hyperinsulinemia, and insulin resistance. The expression of the ATP-sensitive potassium (KATP) channels in the β cells was reported to be reduced.

**Objective:**

To define at the cellular level the role of the KATP channel function for the altered insulin secretion of NZO mice.

**Methods:**

We measured the plasma membrane potential, KATP channel currents, cytosolic Ca^2+^ concentration ([Ca^2+^]_i_), and insulin secretion of NZO mouse islets and β cells in comparison with those of metabolically healthy NMRI mice.

**Results:**

The potassium current of NZO β cells had half the extent of that of NMRI β cells and remained so in the presence of the KATP channel opener, diazoxide. The KATP channel blocker, tolbutamide, reduced the currents to the same low level. At low glucose and in the presence of diazoxide, the plasma membrane potential of NZO β cells was less polarized, and consequently, [Ca^2+^]_i_ was higher in NZO β cells than in NMRI β cells. The depolarization and [Ca^2+^]_i_ increase by tolbutamide were not different. Insulin secretion of the NZO islets was higher than that of NMRI islets at low glucose and in the presence of diazoxide, but was lower than that of NMRI islets in the presence of tolbutamide and of high glucose.

**Conclusion:**

The higher insulin secretion at basal glucose, but not the lower stimulated secretion of NZO islets, is at least partly due to the reduced expression of the KATP channels.

Obesity with insulin resistance is the most important risk factor for the development of type 2 diabetes; however, the majority of obese people do not become diabetic [1]. The role of insufficient insulin secretion as an independent contributing factor for the development of type 2 diabetes was suggested by the observation that glucose-tolerant first-degree relatives of patients with type 2 diabetes had a significantly smaller secretory response than appropriate controls [2-4]. Furthermore, the majority of diabetes susceptibility genes identified in genome-wide association studies were associated with functions of the pancreatic β cell [5].

Thus, for a rodent model of type 2 diabetes to come close to the spectrum of the human disease, it has to combine obesity with defective insulin secretion on a polygenic basis. These criteria are fulfilled by the New Zealand obese (NZO) mouse [6, 7]. The NZO mouse displays fasting hyperglycemia, hyperinsulinemia, hepatic and peripheral insulin resistance, and, later on, defective insulin secretion [8, 9].

In NZO mice, the development of overt type 2 diabetes is limited to male animals, as females of this strain are protected from developing type 2 diabetes. This protective effect against the development of type 2 diabetes in female NZO mice is partly due to the protective effect of estrogen, which leads to reduced insulin resistance and decreased sensitivity of β cells to glucolipotoxic influence [10-12].

The insulin secretion of NZO mice is similar to the impaired insulin secretion of human type 2 diabetes in that the basal insulin secretion rate is elevated and the glucose-stimulated secretion rate is diminished [13-15]. Thus, the dynamic range is markedly reduced compared with metabolically healthy rodents, such as the NMRI mouse [16].

Observations by Andrikopoulos and colleagues suggested that the reduced expression level of plasma membrane ATP-sensitive potassium (KATP) channels is responsible at least in part for the defective secretory response to increases in the ambient glucose concentration [17]. Specifically, it was suggested that the diminished early-phase response to glucose in the IV tolerance test would result from this genetic trait [17]. However, the secretory responses to glucose stimulation of NZO mice, Bl6 mice, and transgenic NZO mice with near-normal KATP channel expression did not correlate with their respective responses to the stimulation by the blocker of KATP channels, tolbutamide [17].

Thus, the relation between the KATP channel activity and the altered kinetics of insulin secretion in NZO mice appears less clear-cut than proposed. To more precisely characterize the relation between KATP channel activity and insulin secretion, we measured the following parameters in isolated islets and single islet cells: KATP channel currents, plasma membrane potential, cytosolic Ca^2+^ concentration ([Ca^2+^]_i_), and insulin secretion. As control, these parameters were also measured in β cells and islets from NMRI mice, a metabolically healthy outbred mouse strain with well-characterized signal transduction of glucose-induced insulin secretion, in particular the metabolic-electrical coupling [18, 19].

Our observations suggest that the altered KATP channel function in NZO β cells is primarily responsible for the increased basal insulin secretion level.

## Materials and methods

### Chemicals

Diazoxide, nystatin, tolbutamide, collagenase P (Roche) and the cell culture medium RPMI 1640 (without glucose) were from Sigma-Aldrich (Taufkirchen, Germany). Fetal calf serum (FCS Gold ADD) was obtained from Bio & Sell (Nürnberg-Feucht, Germany), Fura-2 LeakRes (AM) was purchased from Teflabs (Austin, TX, USA), BSA (fraction V), and all other reagents of analytical grade were from E. Merck (Darmstadt, Germany).

### Islet isolation and tissue culture

Islets were isolated from the pancreas of female NZO mice or female NMRI mice (7-14 weeks old, fed ad libitum) by injection of 3 mL of a collagenase solution (0.75 U/mL) into the bile duct and incubation of the excised pancreas in a water bath for 9.5 minutes. After shaking by hand for 1 minute, the islets released from the exocrine tissue were hand-picked under a stereomicroscope. Islets were either directly used for the measurement of insulin secretion or cultured for 22 ± 1 hours. Single islet cells were obtained by incubation of the islets for a total time of 11 minutes in a Ca^2+^-free Krebs-Ringer (KR) medium and vortex-mixing for 1 minute every 2 minutes. Single islet cells and islets were cultured in RPMI 1640 with 5 mM glucose (single cells in 10 mM for the first 2 hours) and 10% fetal calf serum in a humidified atmosphere of 95% air and 5% CO_2_ at 37 °C. Single cells were cultured for up to 2 days. The mice were from local colonies, SUR1 knockout (KO) mice [20] were originally obtained from Lydia Aguilar-Bryan.

### Measurement of insulin secretion

For each experimental set, 10 size-matched islets, either freshly isolated or 1-day-cultured, were preincubated in a HEPES-buffered KR medium for 1 hour containing 1 mM glucose. This was followed by a 1 hour incubation in KR medium containing 1 mM or 20 mM glucose or 1 and 20 mM glucose in combination with either 250 µM diazoxide or 500 µM tolbutamide. The KR medium was saturated with 95% O_2_ and 5% CO_2_ and contained (mM): NaCl 118.5, KCl 4.7, CaCl_2_ 2.5, KH_2_PO_4_ 1.2, MgSO_4_ 1.2, NaHCO_3_ 20, HEPES 10, and BSA 0.2% w/v. The insulin content of the supernatant was determined by ELISA according to the manufacturer’s protocol (Mercodia, Uppsala, Sweden).

### Measurement of the cytosolic Ca^2+^ concentration

Cultured single islet cells were loaded with 2 µM Fura-2 LeakRes (acetoxymethyl ester) by incubating for 20 minutes at 32 °C in KR medium containing 5 mM glucose. Cover slips with attached cells were then inserted in a temperature-controlled perifusion chamber (35 °C) on the stage of a Zeiss Axiovert 135 microscope equipped with Zeiss Fluar objectives (40×, 1.3 NA). The cells were perifused with KR medium saturated with 95% O_2_ and 5% CO_2._ The fluorescence (excitation at 340 or 380 nm, dichroic separation at 410 nm, emission 510 ± 40 nm) was recorded with a cooled CCD camera (Pursuit, Diagnostics Instruments, Sterling Heights, MI, USA) and evaluated using Visiview software (Visitron, Munich, Germany).

### Electrophysiological recordings

Plasma membrane currents and the plasma membrane potential of 1- or 2-day-cultured single islet cells were measured using the perforated-patch configuration [21]. Pipettes were pulled from borosilicate glass (2 mm o.d., 1.4 mm i.d., Hilgenberg, Germany) by a 2-stage vertical puller (HEKA-Electronics, Lambrecht, Germany) and had resistances between 3 and 5 MΩ when filled with solution. The measurements of the membrane potential and membrane currents were performed using an EPC 7 patch-clamp amplifier (HEKA-Electronics, Lambrecht, Germany) and the FetchEx mode of pClamp 6.03 software (Axon Instruments, Foster City, CA, USA). After additional low-pass filtering by a 4-pole Bessel filter at 2 kHz, the data were stored on a hard disk and analyzed offline using GraphPad Prism 10 software (GraphPad, LaJolla, CA, USA). A slow bath perfusion system was used; all experiments were performed at room temperature (20-22 °C). To elicit potassium currents hyper- and depolarizing steps of 10 mV around a holding potential of −60 mV were used [22]. For the measurements of the membrane potential in the perforated patch configuration the pipette solution contained (mM): 10 KCl, 10 NaCl, 70 K_2_SO_4_,7 MgCl_2_, and 5 HEPES, pH 7.15 plus 125 µg/mL of the pore-forming agent nystatine (2.5% dimethyl sulfoxide final concentration). The extracellular solution contained (mM): 140 NaCl, 5.6 KCl, 1.2 MgCl_2_, 2.6 CaCl_2_, 10 HEPES, and 1 glucose, pH 7.4.

### Immunohistochemistry and immunofluorescence

Pieces of pancreas that were not used for collagenase digestion were fixed in 4% phosphate-buffered formaldehyde for 24 hours. Fixed tissues were then embedded in paraffin according to standard procedures, and sections of 4 μm were prepared. To stain the SUR1 protein, the sections were incubated overnight at 4 °C in the dark with anti-SUR1 antibody (mouse monoclonal, 1: 500; Thermo Fisher, RRID: AB_2735378) after treating with mouse-on-mouse IgG blocking solution (Thermo Fisher, Schwerte, Germany). The detection was performed using antimouse histofine simple stain MAX PO universal immunoperoxidase polymer (Nichirei Biosciences, Tokyo, Japan) and diaminobenzidine, followed by counterstaining with hematoxylin. Insulin and glucagon were labeled by overnight incubation at 4 °C in the dark with antibodies against glucagon (rabbit; 1:250; Cell Marque, Rocklin, CA, USA; RRID: AB_1158352) and insulin (mouse monoclonal; 1:5000; Sigma-Aldrich, St. Louis, MO, USA; RRID: AB_260137). For fluorescence detection, Alexa Fluor 488 (goat antirabbit antibody, 1:500, Jackson ImmunoResearch, West Grove, PA, USA) and Rhodamine Red-X (goat antimouse antibody, 1:200, Jackson ImmunoResearch) were used. Nuclei were stained with DAPI (SeraCare Milford, MA, USA). Transmitted light and fluorescence images were acquired by a Nikon Eclipse Ni-E upright microscope equipped with a CFI Plan Apo 60×/1.42 N.A. objective and a DS-Fi3 Color Camera and NIS elements AR 6 software (Nikon Instruments, Düsseldorf, Germany). For the quantitative evaluation of the immunohistochemical micrographs, the PyCreas program [23] was used. After defining the islet size, the number and distribution of above-threshold pixels in this area were registered.

### Statistics

GraphPad Prism 10 software (GraphPad, LaJolla, CA, USA) was used for statistical calculations and nonlinear curve fitting. If not stated otherwise, “significant” refers to *P* < .05, the specific statistical analyses are given in the figure legends.

## Results

**Figure 1:**
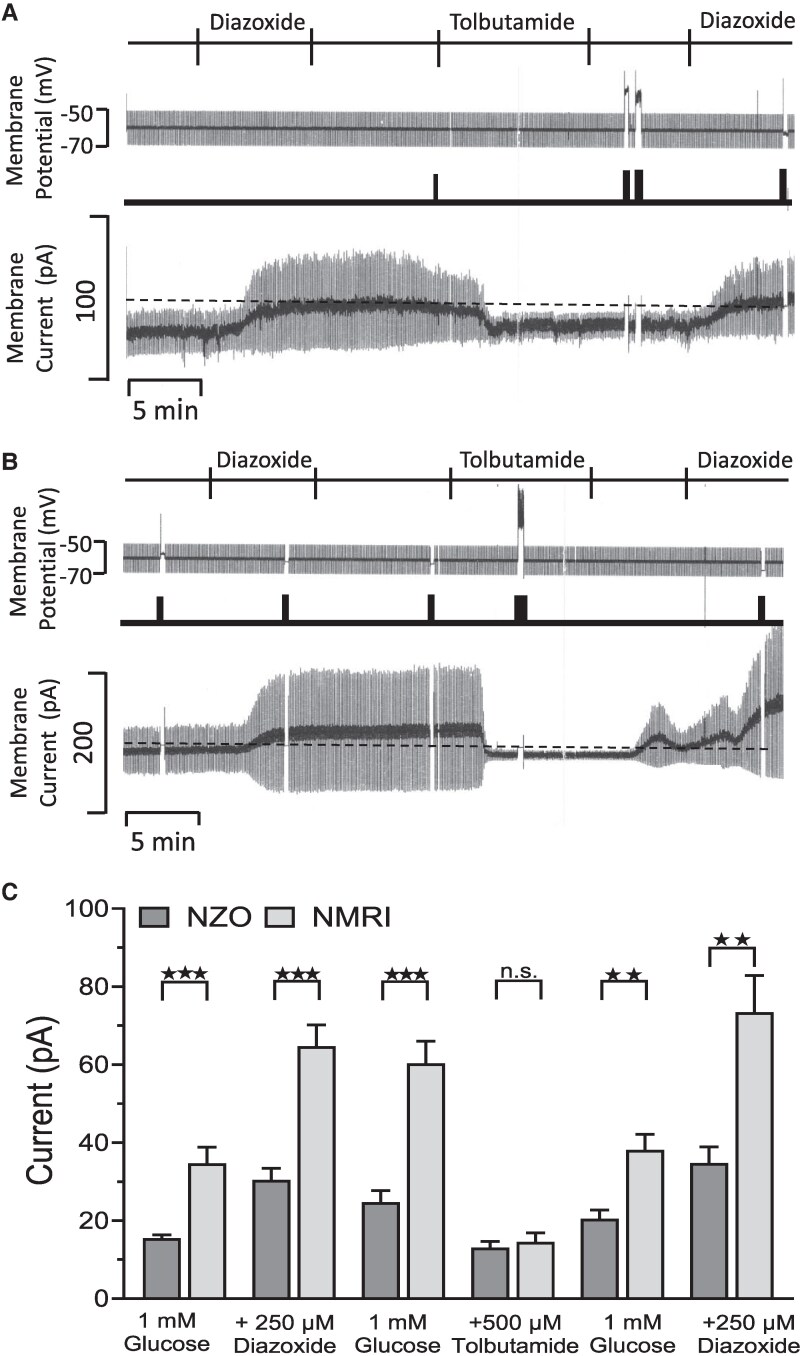
Changes of the β cell membrane currents of NZO mice (A) or NMRI mice (B) in response to 250 µM diazoxide and 500 µM tolbutamide. Original traces of single experiments. β cells were perifused with an extracellular solution containing 1 mM glucose. The currents were elicited by hyper- and depolarizing steps of 10 mV from a holding potential of −60 mV. The zero-current level is indicated by the dashed black line. The membrane potential was verified during short intermittent phases of current clamp, as indicated by the black boxes. (C) Quantitative comparison of the effects of 250 µM diazoxide and 500 µM tolbutamide on the plasma membrane currents of NZO (dark gray bars) and NMRI (light gray bars) β cells in the presence of 1 mM glucose. The data were compared using multiple unpaired *t* tests. Mean ± SEM of 8 (NZO) or 10 (NMRI) experiments. ** and *** indicate *P* < .01 and *P* < .001, respectively. Abbreviations: n.s., nonsignificant; NZO, New Zealand obese.

At 1 mM glucose the currents evoked by hyper- and depolarizing steps of 10 mV were about 50% smaller in NZO than in NMRI β cells (Fig. 1A and 1B). The addition of 250 µM diazoxide practically doubled the current amplitudes with either preparation, and washout slowly diminished the current amplitude (Fig. 1A and 1B). The exposure to 500 µM tolbutamide quickly reduced the current amplitudes, and after 5 minutes, the currents of NZO and NMRI β cells were no longer significantly different (Fig. 1C). Washout of tolbutamide reestablished the original difference between the currents of NZO and NMRI β cells, and renewed exposure to diazoxide led to the same functional consequences as the initial exposure (Fig. 1C).

**Figure 2:**
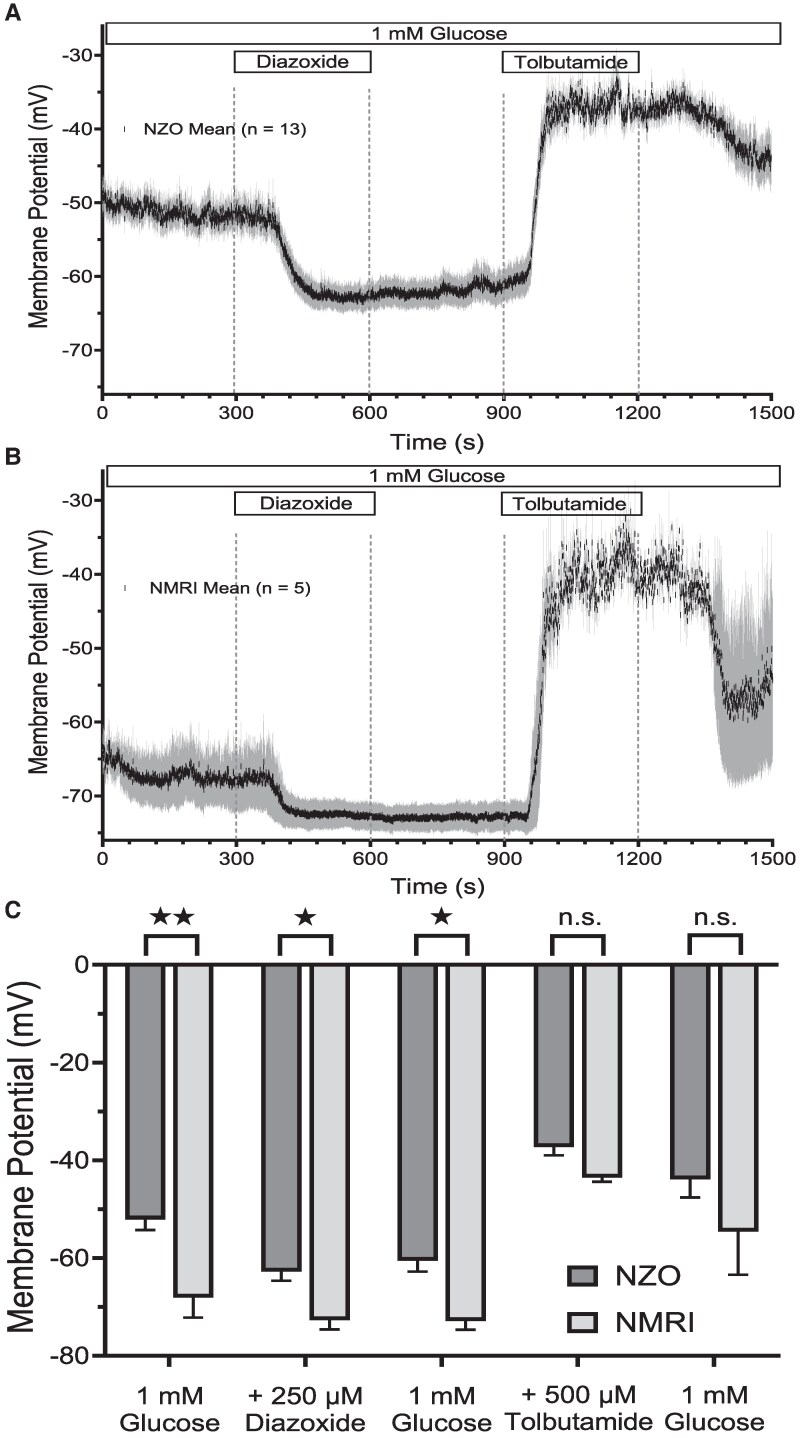
Changes of the β cell membrane potential of NZO mice (A) or NMRI mice (B) in response to 250 µM diazoxide and 500 µM tolbutamide. β cells were perifused with an extracellular solution containing 1 mM glucose. After the establishment of a steady state, 250 µM diazoxide was added for 5 minutes and washed out for another 5 minutes. Then, 500 µM tolbutamide was added and washed out after 5 minutes. The traces are means ± SEM of 13 (NZO) or 5 (NMRI) experiments. (C) Quantitative comparison of the effects of 250 µM diazoxide and 500 µM tolbutamide, as shown in Panels A and B. The data of NZO (dark gray bars) and NMRI (light gray bars) β cells were compared at time points of 300, 600, 900, and 1200 seconds, respectively, by multiple unpaired *t* tests. * and ** indicate *P* < .05 or *P* < .01, respectively. Abbreviations: n.s., nonsignificant; NZO, New Zealand obese.

In the presence of 1 mM glucose, the resting plasma membrane potential was −51.9 ± 2.1 mV for NZO β cells and −68.6 ± 4.1 mV for NMRI β cells (Fig. 2A and 2B). The addition of 250 µM diazoxide hyperpolarized the NZO β cells to −62.7 ± 1.8 mV (Fig. 2A and 2C) and that of NMRI β cells to −72.8 ± 1.8 mV (Fig. 2B and 2C). Washout of diazoxide for 5 minutes slightly depolarized the NZO β cells, but had no effect on NMRI β cells. The exposure to 500 µM tolbutamide led to a fast and strong depolarization with either preparation. After 5 minutes of exposure to tolbutamide, the membrane potential of NZO β cells was no longer significantly different (−37.3 ± 1.7 mV for NZO vs −43.5 ± 0.9 mV for NMRI β cells) (Fig. 2C). In contrast to NZO β cells, NMRI β cells showed a slow oscillatory pattern of the depolarized potential (Fig. 2B). Upon washout of tolbutamide, NZO islet cells repolarized much more slowly than NMRI islet cells (Fig. 2A and 2B).

**Figure 3:**
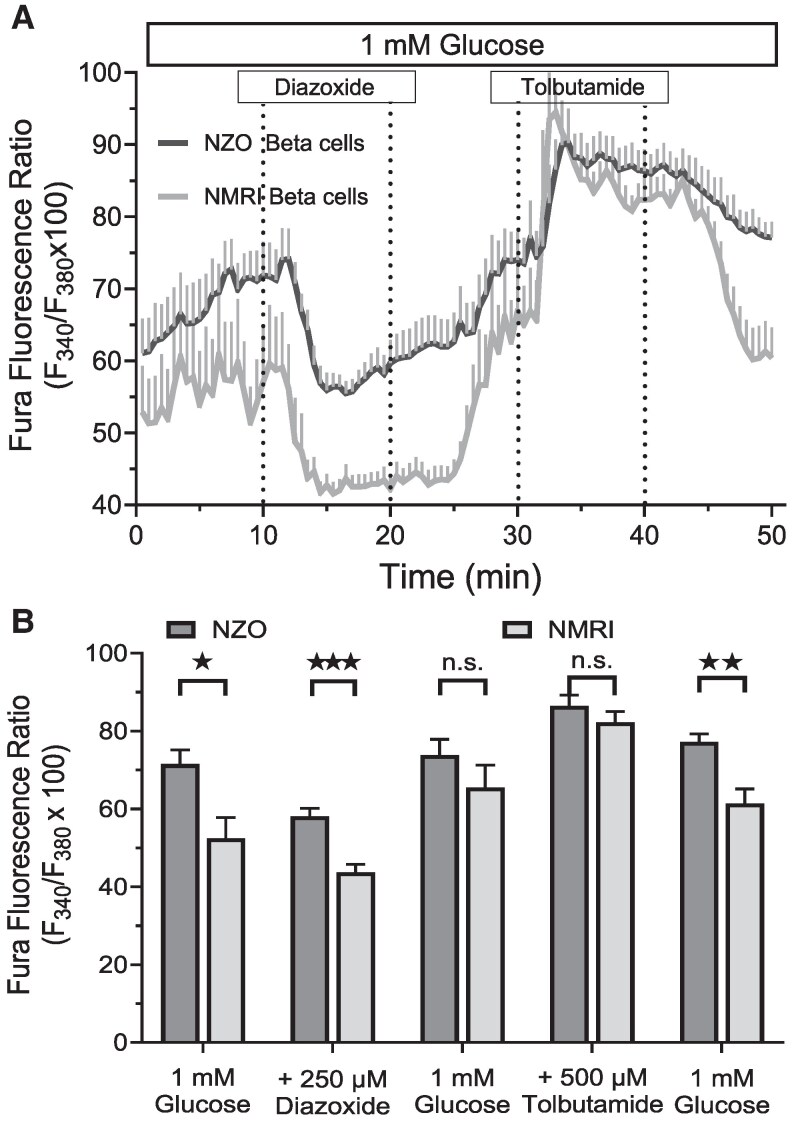
Changes of the [Ca^2+^]_i_ of single β cells from NZO mice or NMRI mice in response to 250 µM diazoxide and 500 µM tolbutamide. (A) Fura 2-loaded β cells from NZO mice (dark gray trace) or NMRI mice (light gray trace) were perifused with Krebs-Ringer medium containing 1 mM glucose. At 10 minutes into the registration, 250 µM diazoxide was added and washed out after 10 minutes. After a washout phase of 10 minutes, 500 µM tolbutamide was added and washed out after 10 minutes. The traces are means ± SEM of 7 (NZO) or 6 (NMRI) experiments. (B) Quantitative comparison of the effects of 250 µM diazoxide and 500 µM tolbutamide, as shown in Panel A. The data of NZO (dark gray bars) and NMRI (light gray bars) β cells were compared at time points 10, 20, 30, 40, and 50 minutes, respectively, by multiple unpaired *t* tests. *, **, and *** indicate *P* < .05, *P* < .01, *P* < .001, respectively. Abbreviations: [Ca^2+^]_i_, cytosolic Ca^2+^ concentration; NZO, New Zealand obese; n.s., nonsignificant.

One-day-cultured islet cells loaded with Fura 2/AM were used to monitor the changes of the free [Ca^2+^]_i_ upon opening and closing the KATP channels (Fig 3A and 3B). At 1 mM glucose, NZO islet cells had a moderately, but significantly higher [Ca^2+^]_i_ than NMRI islet cells. The perifusion with 250 µM diazoxide diminished the [Ca^2+^]_i_ of NZO islet cells as well as that of NMRI islet cells. However, the minimal [Ca^2+^]_i_ values established by diazoxide were significantly higher in NZO islet cells than in NMRI islet cells (Fig. 3B). While the [Ca^2+^]_i_ of the NMRI cells remained stable at low values during the entire exposure to diazoxide, the ratio values of the NZO cells slowly increased (Fig. 3A). After 6 minutes of diazoxide washout, the [Ca^2+^]_i_ values of both cell types increased, and the preexposure level was reached just before the addition of tolbutamide. Tolbutamide (500 µM) strongly raised the [Ca^2+^]_i_ of NZO and NMRI β cells. The [Ca^2+^]_i_ increase of the NMRI cells was steeper and produced the typical initial overshoot (Fig. 5), but thereafter the [Ca^2+^]_i_ values were not significantly different (Fig. 3B). Upon washout of tolbutamide, the slower decrease of the [Ca^2+^]_i_ in the NZO cells was remarkable, which resulted in a significant difference similar to the beginning of the registration.

**Figure 4:**
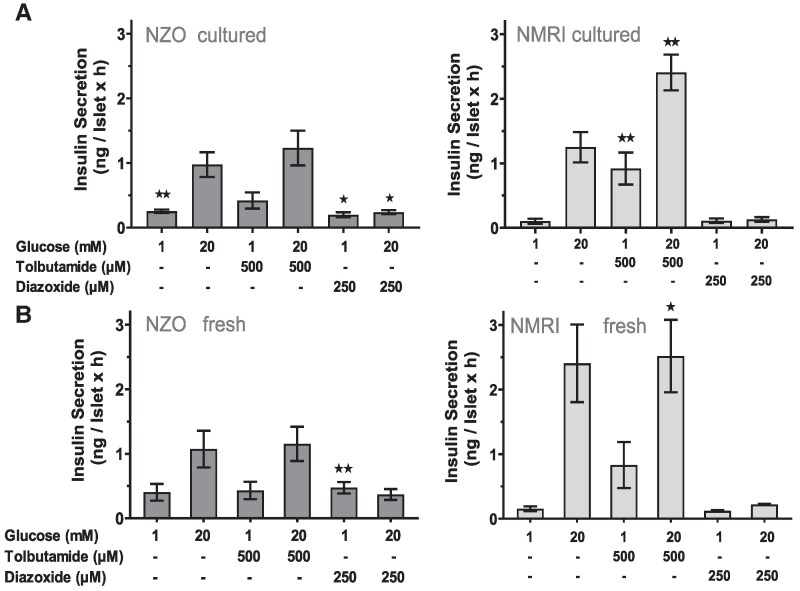
Insulin secretion of 1-day-cultured islets (A) or freshly isolated islets (B) in response to the addition of diazoxide, tolbutamide, or a stimulatory glucose concentration. Islets from NZO mice (dark gray bars) or NMRI mice (light gray bars) were statically incubated with 1 mM or 20 mM glucose or 1 and 20 mM glucose in combination with 250 µM diazoxide or 500 µM tolbutamide. The values for NZO islets were compared with the corresponding values for NMRI islets using Mann-Whitney tests. * and ** indicate *P* < .05 and *P* < .01, respectively. Data are means ± SEM of 5 to 8 experiments. Abbreviation: NZO, New Zealand obese.

At 1 mM glucose, the insulin secretion by cultured NZO islets was significantly higher than the insulin secretion of cultured NMRI islets (Fig. 4A). The additional presence of 500 µM tolbutamide reversed this relation: the secretion by NMRI islets was nearly twice as high as that by NZO islets. With either type of islets, the additional presence of diazoxide did not decrease the insulin secretion below the level established by 1 mM glucose alone. But again, the secretion by NZO islets was significantly higher than that by NMRI islets (Fig. 4A). At 20 mM glucose, cultured NZO islets secreted slightly, but not significantly less insulin than cultured NMRI islets. In the additional presence of tolbutamide, the difference became significant; NMRI islets secreted nearly twice as much insulin as the NZO islets. In the presence of 20 mM glucose, diazoxide strongly reduced the secretion by either islet type to a similar level as in the presence of 1 mM glucose. Again, the secretion by NZO islets was significantly higher than that by NMRI islets. In principle, the same pattern was observed with freshly isolated islets (Fig. 4B). Under each condition, the difference between NZO and NMRI islets, as seen with cultured islets, was reproduced. Because of the larger data scatter, however, only the secretion of NZO islets in the presence of diazoxide and of NMRI islets in the presence of 20 mM glucose and tolbutamide were significantly higher than the secretion of the respective counterparts.

**Figure 5:**
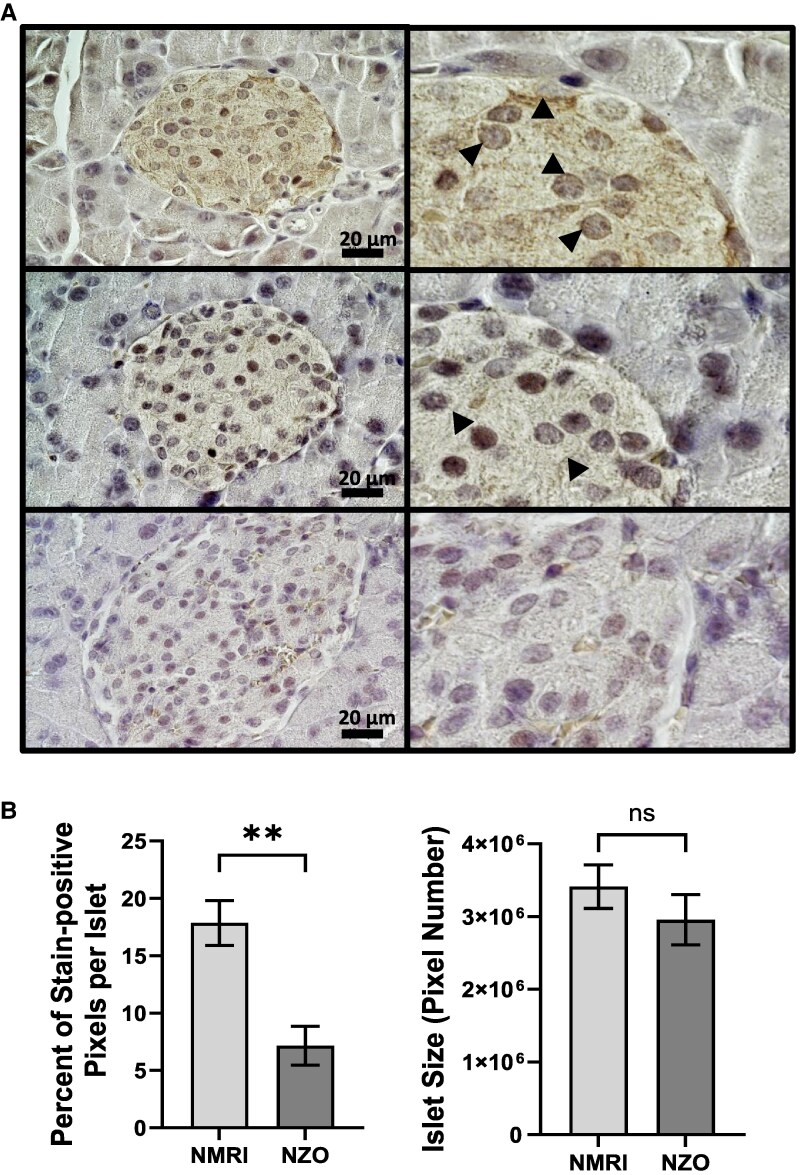
(A) Immunohistochemical staining of the SUR1 subunit of the KATP channel in islet sections from NMRI mice (upper micrographs), NZO mice (middle micrographs), and SUR1 knockout mice (lower micrographs). In the left micrographs, the brownish coloring of the islet decreases in the sequence NMRI > NZO > SUR1 knockout. The surrounding exocrine tissue remained unlabeled. The right micrographs are 3-fold magnified details of the respective image to the left. In addition to the cytosolic labeling, the more intense labeling of the plasma membrane (NMRI and NZO) and of the perinuclear region (NMRI) becomes visible (arrowheads). (B) Quantitative comparison of the staining intensity of NZO and NMRI islets. The values of the NZO islets (n = 17) were compared with the corresponding values of NMRI islets (n = 28) by the Mann-Whitney test (left panel). The size of these islets was also compared by the Mann-Whitney test (right panel). Data are means ± SEM. ** indicates *P* < .01, respectively. Abbreviations: KATP, ATP-sensitive potassium; n.s., nonsignificant; NZO, New Zealand obese.

**Figure 6:**
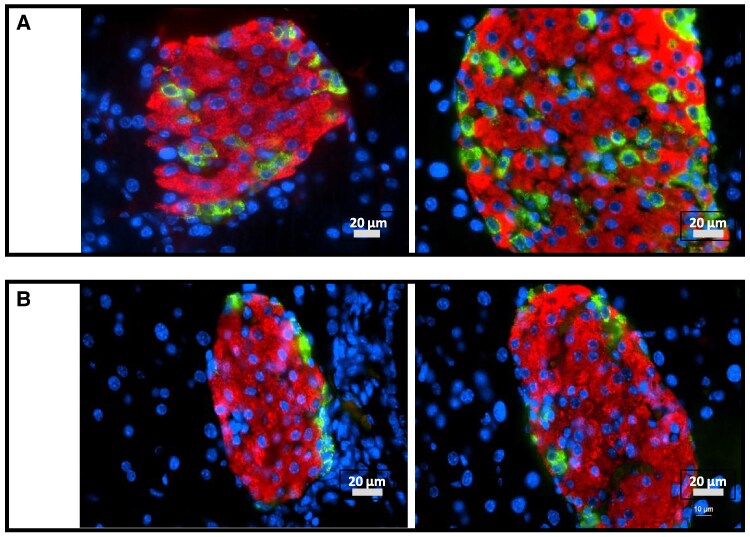
Immunofluorescent staining of α cells and β cells in islets from NZO islets (A) and NMRI islets (B). The α cells are labeled by green fluorescence, and the β cells by red fluorescence; nuclei appear blue. Both in small (left panel) and in large (right panel) islets from NZO mice, α cells are present in the islet center as well as in the periphery, whereas in small (left panel) and in large (right panel) NMRI islets, α cells are only present at the periphery. Typical observation for 7 islets each. Abbreviation: NZO, New Zealand obese.

The premise that NZO β cells express KATP channels to a lower level than the NMRI β cells was verified by immunohistochemistry. The specificity of the used SUR1 antibody was evident from the lack of staining of SUR1 KO islets (Fig. 5A). The staining intensity of NZO islets was higher than that of SUR1 KO islets, but significantly lower than the intense staining of NMRI islets (Fig. 5A and 5B). Another difference between NZO and NMRI islets that may contribute to the different pattern of secretion is the distribution of α cells. While the α cells of NMRI islets were localized at the islet periphery, the α cells of NZO islets were also present in the islet center (Fig. 6A and 6B).

## Discussion

The observations in this investigation provide a logical explanation for a characteristic feature of dysregulated insulin secretion in NZO mice, which is a valid model of human type 2 diabetes: the elevated secretion rate at nonstimulatory glucose concentrations.

At 1 mM glucose, the potassium current of NZO β cells was about half the size of that of NMRI β cells. Diazoxide markedly increased the current in either cell type, but the relation between NZO and NMRI cells remained practically unchanged. The likely explanation for these observations is that the number of channels that can be opened by diazoxide is different. This concurs with the earlier finding that the Abcc8 (SUR1) allele in the NZO genome contains a mutation in the promoter region, which leads to the reduced expression level of SUR1 and, consequently, of KATP channels in NZO β cells [17]. This property of NZO β cells could be confirmed here by the lower immunohistochemical staining of SUR1 in NZO islets than in NMRI islets. As reported earlier [24], SUR1 staining was not restricted to the plasma membrane but was also present in the cytoplasm, most likely caused by the presence of insulin granules and, for unknown reasons, the nuclear membrane [25, 26].

The reduction of the potassium currents by 500 µM tolbutamide to the same low level in both NZO- and NMRI β cells is also compatible with the lower number of KATP channels in NZO β cells. At this concentration, tolbutamide closes the KATP channel by selectively binding to SUR1, achieving virtually complete closure [27]. The small size of the residual current in either cell type shows that KATP channels are by far the predominant contributor to the potassium current, as is typical for pancreatic β cells [18].

Given the dominant role of the KATP channels in the β cells, it is logical that the differences in the KATP channel currents were reflected by differences in the membrane potential. At 1 mM glucose the NZO β cells were less polarized by about 17 mV. This difference comes close to the depolarizing effect of KATP channel closure [28], but was not associated with action potential spiking in NZO β cells. The ability of diazoxide to hyperpolarize either cell type fits with its ability to increase the potassium currents. Even though NMRI currents were still double the size of the NZO currents under this condition, the difference between the membrane potentials decreased to about 10 mV. This is most likely due to the membrane potential of the NMRI β cell approaching the potassium equilibrium potential [29]. The closure of the KATP channels by tolbutamide led to depolarized states in NZO- and NMRI β cells, which were no longer significantly different, corresponding to the comparable residual currents during tolbutamide exposure.

The higher [Ca^2+^]_i_ of NZO than NMRI β cells at 1 mM glucose and at 1 mM glucose plus diazoxide is consistent with their less polarized membrane potential under these conditions. Similarly, SUR1 ko β cells had higher [Ca^2+^]_i_ levels at 1 mM glucose than control β cells [30]. Even though no action potentials were visible, the increased level of [Ca^2+^]_i_ is most likely due to Ca^2+^ influx, as was the case with SUR1 KO β cells [30]. In heterozygous SUR1 KO β cells, which have a 60% reduction of KATP channel density, a left shift of the glucose-induced Ca^2+^ oscillation was noted [31], confirming that a decreased potassium efflux comparable to that of NZO β cells is sufficient to affect the Ca^2+^ signal for exocytosis. The comparable [Ca^2+^]_i_ levels of NZO and NMRI β cells in the presence of tolbutamide, which reflect the similarly depolarized plasma membrane potential, suggest that voltage-dependent Ca^2+^ channels are not functionally different in NZO and NMRI β cells.

The higher insulin secretion of NZO islets than of NMRI at 1 mM glucose and at 1 mM glucose plus diazoxide fits the higher [Ca^2+^]_i_ of NZO islets under these conditions. However, the lower secretion of NZO islets in response to tolbutamide stimulation, both in the presence of 1 and 20 mM glucose, cannot be explained by lower [Ca^2+^]_i_. A desensitization of the exocytotic machinery to Ca^2+^ has been suggested to occur in SUR1 KO because of chronically elevated [Ca^2+^]_i_ [32]. Desensitization of insulin secretion is probably a general response to Ca^2+^ influx caused by depolarization without nutrient stimulation [33].

The pattern of secretory responses of freshly isolated islets corresponded to that of cultured islets. Even though the larger data scatter often impeded statistical significance, the higher secretion of the NZO islets with diazoxide and the lower secretion with tolbutamide were clearly recognizable. The additional measurement of secretion of fresh islets was performed to enable comparison with the results of a recent study where the kinetic secretion of freshly isolated islets and insulin levels during oral glucose tolerance testing of NZO and NMRI mice were characterized [34].

Despite quantitative differences, the overall pattern was qualitatively similar: In contrast to the nonsignificantly lower response of NZO islets to 20 mM glucose here, the perifusion showed a significant 3-fold lower insulin secretion upon glucose stimulation, whereas the basal secretion rate was only slightly higher than that of the perifused NMRI islets [34]. During the oral glucose tolerance testing, however, the insulin concentrations of the NZO mice were much higher than those of the NMRI mice from the beginning and remained so during the entire test period [34]. Thus, the β cell of juvenile or adult NZO mice is not in a state of exhaustion as has been shown earlier by the vigorous response to arginine stimulation [17].

Rather, additional mechanisms must contribute to the inappropriate secretory response to glucose. A possible reason for the high insulin levels in vivo is the increased activity of the amplifying pathway, as observations on SUR1 KO islets suggest [30]. While this is probably a compensatory measure, secondary to the defect of the physiological triggering signal, the reported lower glucose utilization of NZO mice [35] can be regarded as an independent factor leading to a defect in signal recognition and, in consequence, to the defective response to increases in glucose concentration. Furthermore, the metabolic disturbance of the NZO mouse may not only result from dysregulated insulin secretion. The hyperinsulinemia of the NZO mice in vivo is accompanied by a hyperglucagonemia [34], which may be related to the increased presence of α cells throughout the NZO islet, as has been observed recently [23] and could be confirmed here. While pronounced variations in the distribution of α cells exist across different species, apparently without association with metabolic disorders [36], the distribution in NZO mice, which is atypical for rodents, may result from the transdifferentiation of β cells into α cells [37, 38] and thus contribute to the pathophysiology.

The value of the NZO mouse as a model for human type 2 diabetes lies in its combination of obesity with defective insulin secretion on a polygenic basis. Insulin resistance has long been regarded as the driving force behind the development of type 2 diabetes [39]. Overt diabetes would develop when the β cells lose their ability to sustain the compensatory hyperinsulinemia, resulting in the transition from hyperinsulinemia to hypoinsulinemia. In recent years, this view has been questioned [40]. Hyperinsulinemia may be the cause, not the consequence, of obesity and of the insulin resistance associated with it [41]. Damage to the β cell would result from the excitotoxicity associated with high [Ca^2+^]_i_ levels and the metabolic stress resulting from overnutrition [42, 43]. Excitotoxicity, caused by loss-of-function mutations in KATP channels, has been shown to ultimately lead to insufficient insulin secretion even in the absence of obesity [44]. The present observation that the low KATP channel expression level in NZO β cells is the underlying reason for the insulin hypersecretion at low glucose fits this reconsideration of the pathogenetic relation between obesity and insulin secretion.

## Data Availability

Some or all datasets generated during and/or analyzed during the current study are not publicly available but are available from the corresponding author on reasonable request.

## Abbreviations

[Ca^2+^]_i_: cytosolic Ca^2+^ concentration
KATP: ATP-sensitive potassium channel
KR: Krebs-Ringer
n.s.: nonsignificant
NZO: New Zealand obese
SUR: sulfonylurea receptor
